# A Mechanistic, Stochastic Model Helps Understand Multiple Sclerosis Course and Pathogenesis

**DOI:** 10.1155/2013/910321

**Published:** 2013-03-12

**Authors:** Isabella Bordi, Renato Umeton, Vito A. G. Ricigliano, Viviana Annibali, Rosella Mechelli, Giovanni Ristori, Francesca Grassi, Marco Salvetti, Alfonso Sutera

**Affiliations:** ^1^Department of Physics, Sapienza University of Rome, Piazzale Aldo Moro 2, 00185 Rome, Italy; ^2^Centre for Experimental Neurological Therapies (CENTERS), Neurology and Department of Neurosciences, Mental Health and Sensory Organs, S. Andrea Hospital, Sapienza University of Rome, via di Grottarossa 1035-1039, 00189 Rome, Italy; ^3^Department of Physiology and Pharmacology, Sapienza University of Rome, Piazzale Aldo Moro 5, 00185 Rome, Italy

## Abstract

Heritable and nonheritable factors play a role in multiple sclerosis, but their effect size appears too small, explaining relatively little about disease etiology. Assuming that the factors that trigger the onset of the disease are, to some extent, also those that generate its remissions and relapses, we attempted to model the erratic behaviour of the disease course as observed on a dataset containing the time series of relapses and remissions of 70 patients free of disease-modifying therapies. We show that relapses and remissions follow exponential decaying distributions, excluding periodic recurrences and confirming that relapses manifest randomly in time. It is found that a mechanistic model with a random forcing describes in a satisfactory manner the occurrence of relapses and remissions, and the differences in the length of time spent in each one of the two states. This model may describe how interactions between “soft” etiologic factors occasionally reach the disease threshold thanks to comparably small external random perturbations. The model offers a new context to rethink key problems such as “missing heritability” and “hidden environmental structure” in the etiology of complex traits.

## 1. Introduction

Multiple sclerosis (MS) is an immune-mediated disease of the central nervous system with a relapsing-remitting course in the majority of the early stages of the disease [1]. As for other multifactorial diseases, there is no comprehensive overview of the events that lead to the disease. This limits the opportunities provided by the advancements in genetics, immunology, and neurobiology since it is difficult to contextualize each single discovery. The uncertainties in the interpretation of genome-wide association studies (GWAS) reflect, to some extent, this problem. These studies carried the expectation to define the heritable component in multifactorial diseases and, through this, also sketch the nonheritable (environmental) contribution to the phenotype. As largely witnessed by the debate about “missing heritability” in multifactorial diseases, also this powerful approach appears to be in need of interpretative keys as neither genes nor the environment seem to harbour factors that, alone or jointly, are strong enough to explain the disease etiology [2, 3].

Likewise situations are rather common in the physics of nonlinear systems; here the observed large variations are explained through the effects induced by small random perturbations [4–6]. An example is the theory of the Earth's climate: the cooperative effect of a small stochastic perturbation and periodic forcing (variation of astronomical parameters) provides an amplification of the climate response known as “stochastic resonance” that leads to the transition from a temperate climate to an ice-covered Earth state and vice versa [5–7].

Random molecular events can contribute to phenotypic diversity in isogenic populations. Such variation can arise from random fluctuations in gene expression [8, 9], and in fact the term “gene expression noise” is typically used in broad reference to variations among seemingly identical cells experiencing the same environment [10]. Noise in genetic circuits can have profound implications also in multicellular organisms enabling physiological regulation mechanisms, differentiation strategies, and facilitating adaptation and evolution [11], often with a qualitatively different outcome than a deterministic one [12]. As such, these discrete and random fluctuations in the expression of individual genes may also participate in the transition between health and disease by exposing hidden genetic and environmental risk. 

We show that, in MS, transitions between remissions and relapses (i.e., states of relative health and disease, resp.) indeed occur randomly suggesting that stochastic events may contribute to disease pathogenesis. This is described in a model that accommodates the erratic course of the disease and concedes that “soft” heritable and nonheritable perturbations can be amplified over time. The model is based on the equation for a double-well forced by a random perturbation (noise) [13]. We assume that the patient has two states, disease (relapse) and health (remission), and that the barrier to overcome for going from one state to the other is determined by his genetic background and environmental exposures. The small random perturbation is thought to be the inherent variability in gene expression that triggers the erratic sequence of relapses. 

In the likely hypothesis that the mechanisms leading to the transition between remission and relapse recapitulate those that are responsible for the transition between health and disease at onset, time and noise appear as crucial variables that can “amplify” gene-environment interactions and effects until, at one time or the other, the transition towards the disease state occurs in “any” susceptible patient, provided that enough observation time is given. 

## 2. Materials and Methods

### 2.1. Patients Data

We used a dataset of relapses and remissions of 70 patients (28 males and 42 females) with definite MS [14, 15] who were monitored prospectively along the years at the MS Clinic of Sapienza University of Rome (Italy) by four experienced MS neurologists. All patients were free of any disease-modifying therapy (all data are antecedent 1993) and had received short courses of corticosteroids if deemed necessary during relapses. Times to disability end-points were not different between this cohort and published data on the natural history of the disease. The period of interest starts with the first relapse at onset and ends with the last one before the shift to a secondary progressive form. A relapse was defined [14] as the occurrence of new symptoms, the reappearance of former ones, or the worsening of current symptoms of at least 24 hours duration but less than 6 month. Its duration was calculated as the interval between onset of the first symptom or sign and maximum improvement of the last symptom or sign. As part of the standard procedures of specialized MS Clinics, which require to firmly establish the presence or absence of disease activity and progression in order to ensure the most appropriate therapeutic interventions, symptoms were routinely verified during visits at the outpatient service of the MS Clinic. Being this a analysis of anonymous clinical data (collected until 1993, before the institution of ethics committee in Italy in 1998), stored for both clinical and research purposes in the database of the University hospital, ethics committee approval and written informed consent of patients are not needed (http://www.garanteprivacy.it/garante/doc.jsp?ID=1884019).

Each subject has been represented by a sequence of plus one (+1) and minus one (–1) corresponding to the states of relapses and remissions, respectively. For clarity, hereafter these two states are denoted as *no health* and *health*. Events are reported on a weekly scale (shorter exacerbations have been rounded up to one week). 

### 2.2. The Model

First, the dataset is analyzed to study the time evolution of relapses, their time duration, and their statistical distribution. Secondly, a simple model consisting of a mechanistic component and a stochastic perturbation [13] is introduced to represent the main characteristics observed in the clinical histories of patients. The mechanistic component provides the deterministic (predictable) component of the model, while the stochastic perturbation describes an “external stimulus” whose behaviour is intrinsically non-deterministic (random) and that can be analyzed only in probabilistic terms. The latter is usually called “stochastic forcing” since it acts as a force that perturbs the deterministic component, making the model solution unpredictable. 

**Figure 1:**
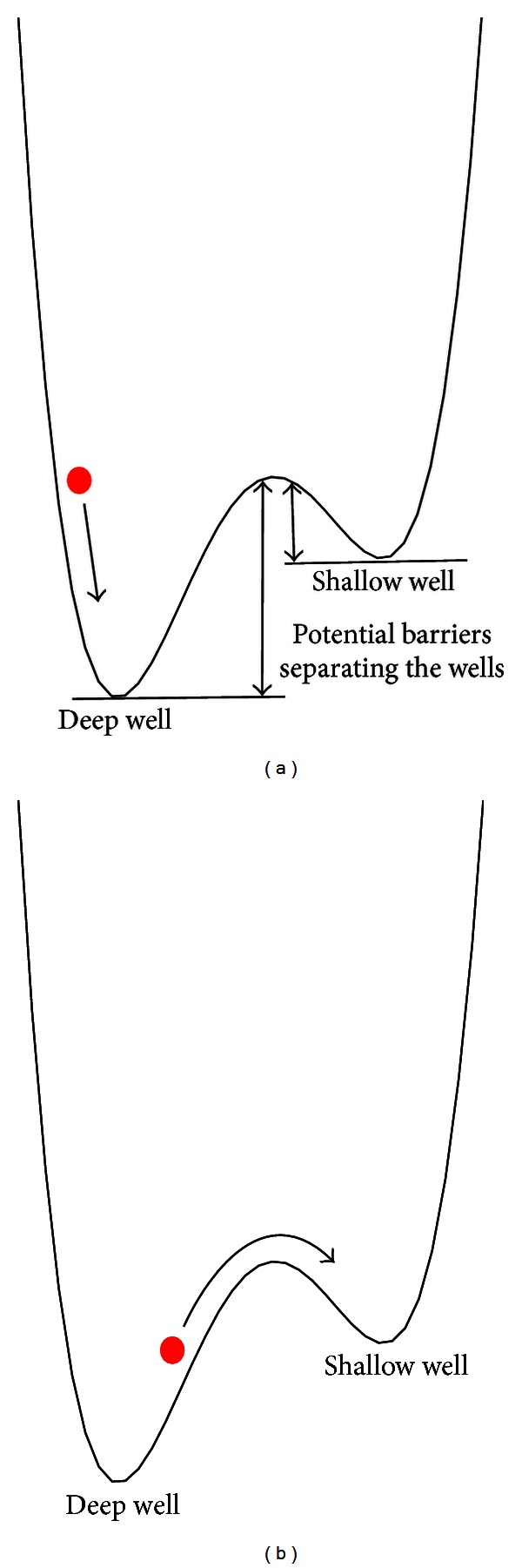
Schematic picture of a particle in a double-well energy potential. (a) The unperturbed particle falls into one of the two wells from its initial position (deterministic case). (b) The particle subjected to a random perturbation jumps from a well to the other randomly (stochastic case).

The underlying hypothesis of the model here proposed is that the health state of a patient in time behaves likewise the motion of a particle of unit mass in a double-well energy potential as schematically illustrated in Figure 1. Now, given any initial position of the particle and if no perturbation is applied on it (i.e., under deterministic conditions), the particle will fall in one of the two potential wells and will remain there for infinitely long time, independent of whether the well is deep or flat (Figure 1(a)). If, instead, there is a strong stochastic perturbation acting on the particle, it will randomly move forth and back between the two wells, independently of their depth. The most interesting situation for us occurs when there is a small/moderate stochastic perturbation acting on the particle: the particle will typically stay for some time in the well it occupies, until the random diffusion drives it over the potential barrier into the other well (Figure 1(b)). It is intuitively clear that the exit from a flat well happens faster than the exit from a deep well. 

In the phenomena of diffusion, like the one just described, the random perturbation is represented by the Wiener process [16], which consists of a normally distributed white noise (i.e., the amplitude of the noise is normally distributed with given mean and standard deviation, and all frequencies are involved with no time periodicity). 

We note that the noise in physical systems was considered for a long time just as a nuisance that degrades the signal. Later on it was found that, in complex (nonlinear) processes, as the two-state process here considered, it may introduce a longitudinal dependence corresponding to the erratic switching between the steady states that, otherwise, would be persistent forever [5]. This property of noise should be distinguished from the mechanism of “stochastic resonance” introduced to explain the cyclic recurrence of ice ages and that is based on the combined effect of noise and a periodic external forcing [6]. In that case, useful applications have been found in physical, technological, and biomedical contexts ([17–20] and references therein).

Coming back to the metaphor particle-patient, the noise may represent small biological variations of random character experienced by the patient at each time. The bottom of the two wells represents the *health* and *no health* states, while the height of the wells is the barrier to be overcome for going from one state to the other, here supposed to be determined by the genetic background and environmental exposures of the patient. Only the effect of noise over time can let the patient change his state of health into disease and be subjected to random relapses. 

## 3. Results

### 3.1. Some Evidence from Clinical Data

**Figure 2:**
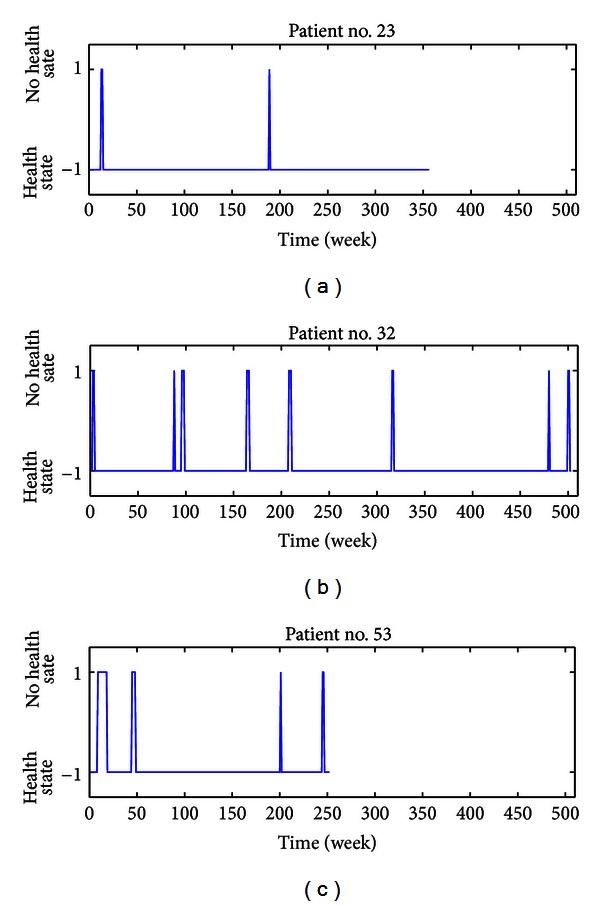
Time evolution of *health* and *no health* states for three sample patients taken from the set of 70 patients. The health state of each patient has been represented by a sequence of plus one (+1) and minus one (−1) corresponding to the states of relapses and remissions, respectively. Events are reported on a weekly scale and the shift to a secondary progressive form of the disease has been excluded.

By analyzing the dataset of 70 patients, some evidence emerges.  Figure 2 shows the time evolution of the health states for three sample patients. The time series suggest the unpredictable nature of relapses (+1 states) followed by periods of relative quiet remission with no new signs of disease activity (–1 states). Also, the time in a health state seems much longer than the one spent in a disease state. It is worth noting that the transition between the two states, by construction, is here represented by a step function (abrupt change of state). Such a choice can be reductive or unrealistic since patients usually report a slow or subacute onset of the relapses. However, this feature does not affect the results presented below.

**Figure 3:**
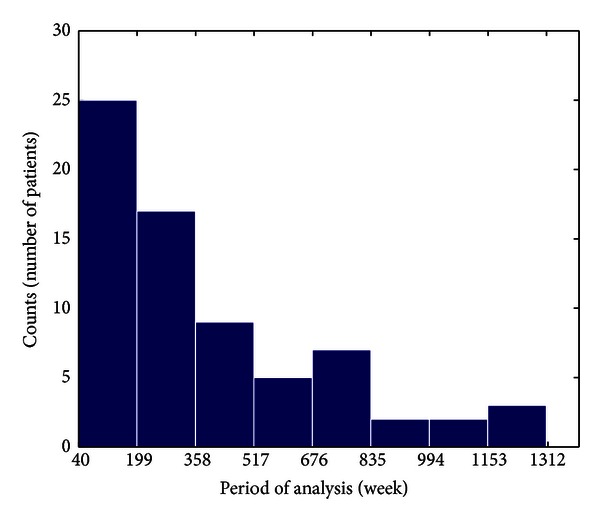
Histogram of the duration of the relapsing-remitting phase in weeks for the 70 selected patients. The duration of the relapsing-remitting phase (i.e., analysis period) varies from one patient to the other, from a minimum of 40 weeks to a maximum of 1311 weeks, following an exponential decaying distribution. For 25 of 70 selected patients (about 36%), the relapsing-remitting phase lasts for about 200 weeks (about 4 years).

As shown in Figure 3, the duration of the relapsing-remitting phase (i.e., period of analysis) varies from one patient to the other, from a minimum of 40 weeks to a maximum of 1311 weeks (about 27 years). As illustrated by Figure 3, for 25 of 70 patients (about 36%) the relapsing-remitting phase lasts for about 200 weeks (about 4 years), and moving towards longer periods the percentage of interested patients quickly decreases to zero (exponential decaying distribution).

**Figure 4:**
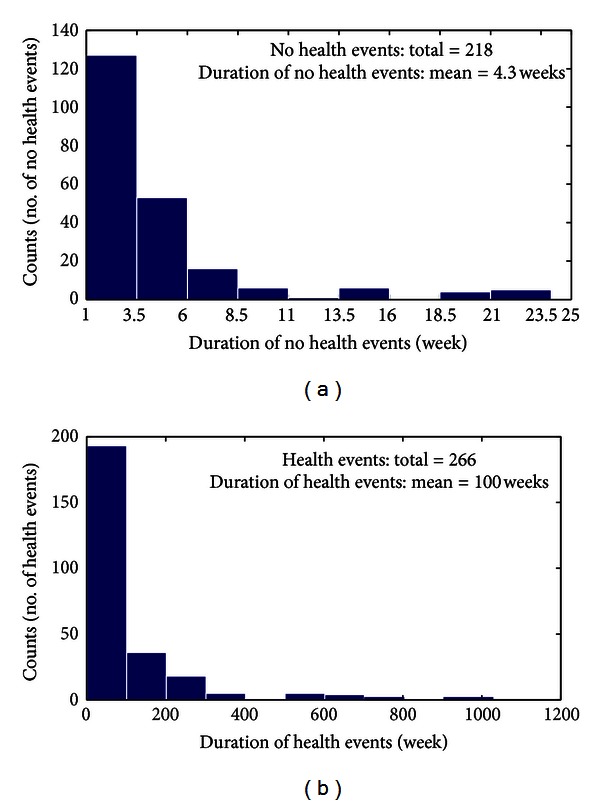
Histograms of the time duration of relapses and remissions for the 70 selected patients. (a) Histogram of *no health* events (+1 states); (b) histogram of *health* events (−1 states). Both figures show exponential decaying distributions with no sign of periodicity (i.e., peak at given duration), suggesting that disease events occur randomly in time. To be noted is the different duration of relapses and remissions (on average 4.3 weeks against about 100 weeks).

Similarly, the distributions of the time duration (or exit time) of relapses and remissions follow exponential decaying behaviours (Figures 4(a) and 4(b)). The mean duration of disease attacks is about 4.3 weeks, with a minimum of 1 week to a maximum of about 24 weeks (rare events). The mean time spent by patients in the *health* state is instead about 100 weeks, with a minimum time interval between two consecutive disease events of a few weeks up to about 1000 weeks in exceptional cases. It is worth noting that the absence of any peak in the distributions at some specific time corroborates what has been noted before for sample patients (Figure 2): disease events occur randomly in time. Moreover, the mean duration of disease events is much shorter than that of remission. 

In the following, we will show how these features can be taken into account in a simple mechanistic model forced by random perturbations. The aim is to contribute to the understanding of the mechanisms that underlie the disease, highlighting the role of noise in a system (patient) that is primarily governed by mechanistic laws. 

### 3.2. The Model: Mechanistic Component

The model to simulate the random transitions between the* health* and *no health* states (–1 and +1, resp., as in clinical data) is here introduced.

Let *x* be the health state of a patient (the position of the particle in our metaphor). For a two-state process, as the one we are interested in, the time evolution of *x* is given by the following equation:

(1) $$\begin{array}{c} dx=x\left(1-\alpha x^{2}\right)dt, \end{array}$$

where *t* is the time, *α* is a control parameter, and *dx* and *dt* are the infinitesimal variation of the health state and of time, respectively. The above equation has three steady (independent on time) states. They are *x*_0_ = 0, $x_{1}=-1/\sqrt{a}$, and $\;x_{2}=+1/\sqrt{a}$. Note that in case *α* = 1, we have *x*_1_ = −1 and *x*_2_ = 1. Now, in agreement with the convention used for clinical data, let *x*_1_ be the state of *health* and *x*_2_ the state of *no health*.

The potential *V*, associated with (1) can be easily computed [6] as,

(2) $$\begin{array}{c} V\left(x\right)=-\int x\left(1-\alpha x^{2}\right)dx=-\frac{1}{2}x^{2}+\alpha \frac{1}{4}x^{4}. \end{array}$$

It can be noted that *V*(*x*) is a symmetric function with respect to a change of sign of the *x* variable (it is the same for *x* greater than zero or *x* less than zero), describing a double-well. 

**Figure 5:**
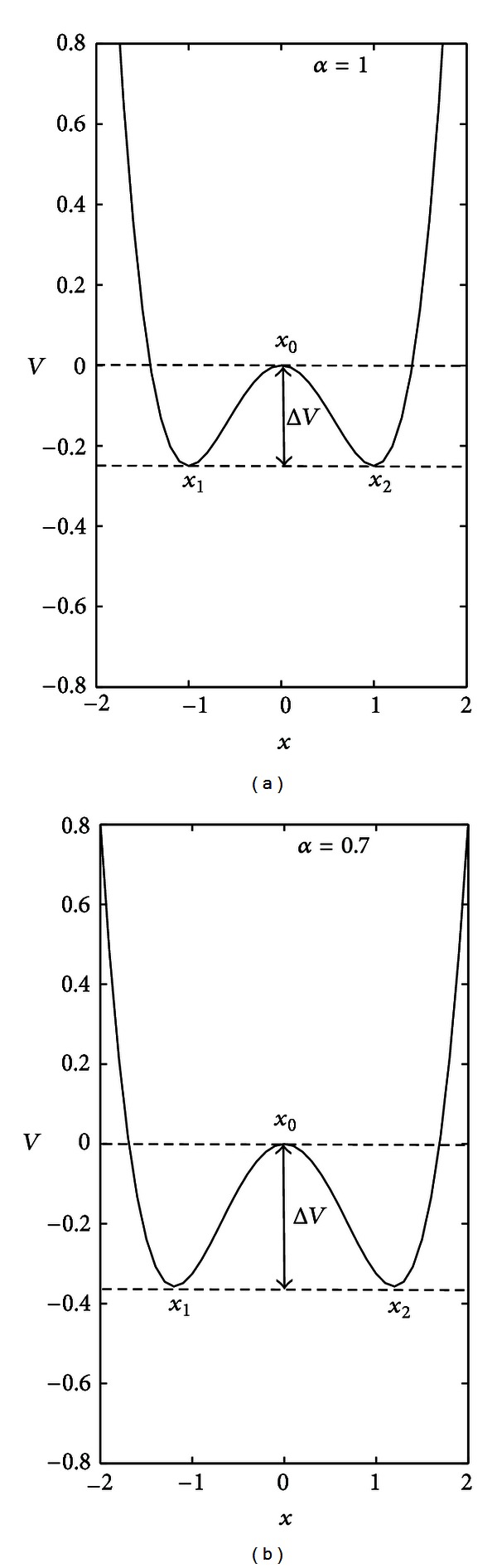
Potential *V* (double-well) as a function of the state variable *x*. The potential *V* associated with the mechanistic model described by (1) is plotted for two values of the parameter *α* controlling the height of the wells: (a) *α* = 1 and (b) *α* = 0.7. The variable *x* represents the health state of a patient (i.e., the position of the particle in our metaphor). The *health* state is denoted by *x*_1_, the *no health* state by *x*_2_, and *x*_0_ is the unstable steady state between them. Δ*V* is the barrier whose height is controlled by the parameter *α*. Dashed lines denote the upper and lower bounds of the potential barrier.

For clarity, we show in Figure 5 the potential *V* as a function of *x* for two values of the control parameter: *α* = 1 and *α* = 0.7. It should be noted that the difference of the potential in *x*_1_ (*x*_2_) and *x*_0_ (denoted by Δ*V* in the figure) is the barrier that must be overcome to jump from one state to the other; it increases when *α* is decreased with respect to the reference value *α* = 1 (the opposite occurs when *α* is greater than 1). In the context of MS, such a barrier is though to be set by the combination of heritable and nonheritable risk.

Thus, according to (1) and (2), we have

- if, by any chance, at initial time the patient is nearby *x*_1_, he will be there forever;
- if, by any chance, at initial time the patient is nearby *x*_2_, he will be there forever;
- if, by any chance, at initial time the patient is nearby *x*_0_, he will have 50% chance to be forever nearby *x*_1_ or *x*_2_.

Now, as shown by clinical data (Figure 2), the patient (*x*) stays a longer time in the *health* state compared to the *no health* state. This feature suggests that the depth of the two wells differs and, in particular, the one associated to the *health* state is deeper than the other, so that it is more difficult to exit from the health condition than from the relapse. In our mathematical model, this implies the introduction of an asymmetry in *V*(*x*), as

(3) $$\begin{array}{c} V\left(x\right)=-\int \left[x\left(1-\alpha x^{2}\right)-\beta \right]dx=-\frac{1}{2}x^{2}+\alpha \frac{1}{4}x^{4}+\beta x, \end{array}$$

where the parameter *β* determines the magnitude and shape of the asymmetry. 

**Figure 6:**
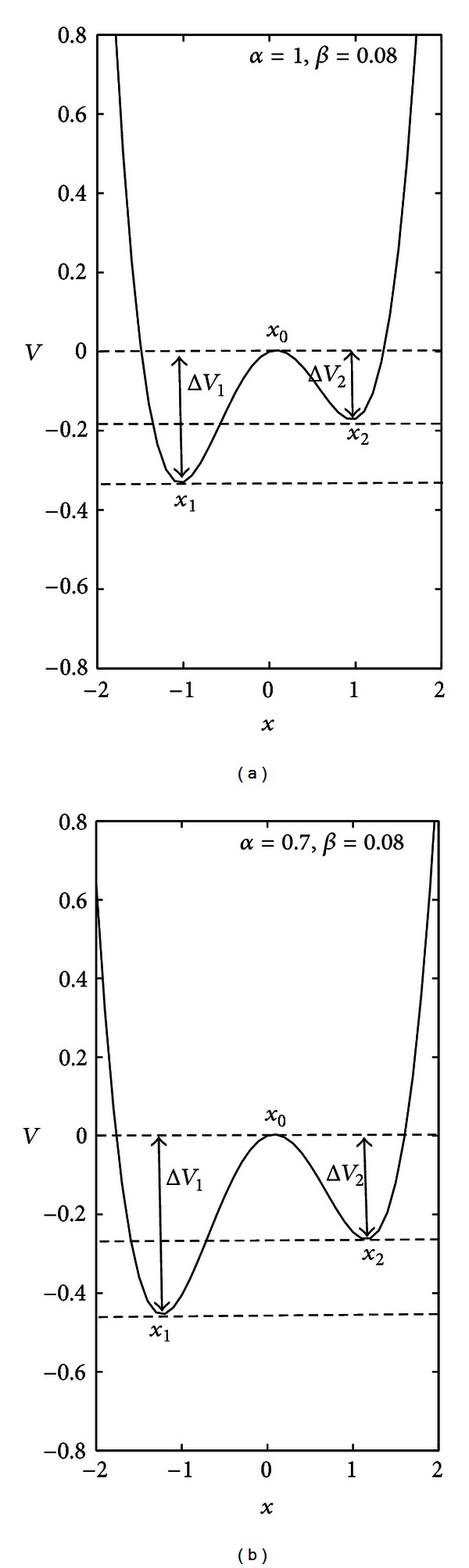
Asymmetric potential *V* as a function of the state variable *x*. The asymmetry in the depth of the wells is obtained setting *β≠* 0 in (3). Here the effect of *β*  (*β* = 0.08) on the shape of the potential *V* is illustrated for the same values of the control parameter *α* used in Figure 5: (a) *α* = 1 and (b) *α* = 0.7. Δ*V*_1_ and Δ*V*_2_ are the barriers between *x*_1_ (*x*_2_) and *x*_0_, respectively. Dashed lines denote the upper and lower bounds of the potential barriers.

Figure 6 illustrates an example of asymmetric double-well (*β* = 0.08) for *α* = 1 and *α* = 0.7. In Figure 6, the barriers are denoted as Δ*V*_1_ and Δ*V*_2_ and are the difference in the potential between the states *x*_1_ and *x*_0_, and *x*_2_ and *x*_0_, respectively. In all the three circumstances above, such barriers separate the states *x*_1_ and *x*_2_, preventing the switching between the wells. Now the question is: can some external random stimuli provide enough energy for diffusing *x* across the barriers, regardless the initial conditions?

### 3.3. The External Random Stimuli

Unless new risk factors are discovered, those identified so far in MS appear unsuitable as deterministic triggers of continuous and random transitions between health and disease, inducing very different disease courses not only in different patients but also in the same patient at different time points. As noise is a source of variability in biological systems, we tried to model it as a driving force. This kind of forcing must be specified through its statistical properties. Let us suppose that on average the noise has zero effect (i.e., its time mean is zero), has a given variance, and it is time decorrelated (i.e., at a given time the value of the stimulus depends only on the previous time): this is the zero order approximation of the statistics of noise (i.e., the Wiener process previously discussed). By introducing such external random perturbation into (1), we have

(4) $$\begin{array}{c} \underset{\begin{array}{c} \text{change}\;\text{of}\;\text{the}\;\text{health}\\ \text{state}\;\text{of}\;\text{a}\;\text{patient} \end{array}}{dx}=\underset{\begin{array}{c} \text{mechanistic}\;\text{component}\\ \left(\text{double-well}\right) \end{array}}{\left[x\left(1-\alpha x^{2}\right)-\beta \right]dt}\;+\underset{\begin{array}{c} \text{external}\;\text{random}\\ \text{stimuli} \end{array}}{\varepsilon^{1/2}dw,} \end{array}$$

where *ε* ^1/2^ *dw* is the noise of variance *ε*. Because of the external random stimuli, this equation has important deviations from its deterministic version described by (1): now the patient can change randomly his state from *health* to *no health* and vice versa.

Averaged over time it is impossible that *x* crosses the barrier since the noise, by construction, has zero mean. However, given the statistical property of the stimulus, in due time, we expect that a sequence of small driving occurrences may be equally signed so that *x* may overcome the barrier and switch from one state to the other. In other words, *x* is harvesting energy from the stimuli for crossing the barrier. For the sake of clarity, the relationship among disease characteristics and model parameters are summarized in Table 1.

As we shall see, this model fits nicely the statistics of the exit times of disease events and explains a few paradoxes encountered in understanding the disease course.

### 3.4. Model Solutions

**Figure 7:**
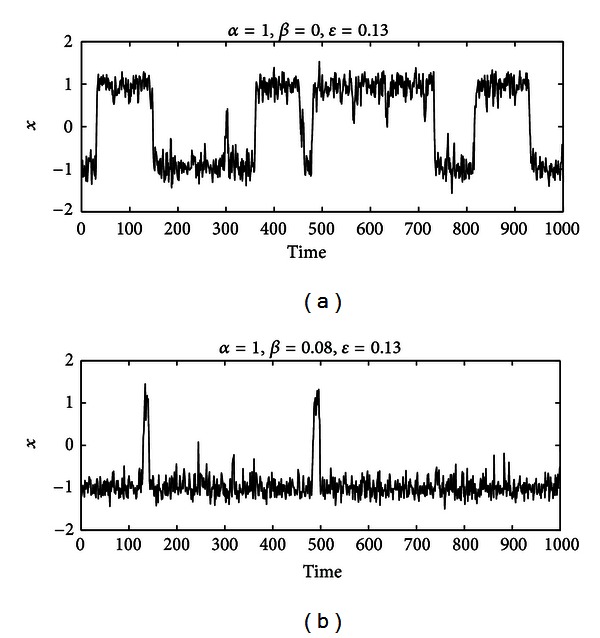
Solutions of the mechanistic, stochastic model. The solutions of (4) as a function of time are shown for (a) the symmetric (*β* = 0) and (b) asymmetric (*β* = 0.08) double-well potential. In both cases, the control parameter is set to *α* = 1, and a stochastic perturbation of the same variance is applied (*ε* = 0.13). Under the effect of the random perturbation, the state variable *x* (i.e., the health state of a patient in our metaphor) jumps erratically from −1 to +1 and vice versa (a). When a small asymmetry is introduced in the potential *V*, the variable *x* still changes erratically its state but spends more time around −1 than around +1 (b). The latter case appears suitable for describing the random occurrences of relapses in MS as observed in clinical data.

To better understand the model, let us look at the solutions of (4) for different values of the parameters. Setting *α* = 1, the solution of (4) as a function of time is shown in Figure 7 for the symmetric and asymmetric double-well cases (*β* = 0 and *β* = 0.08, resp.). According to our metaphor, the patient alternates periods of wellness and disease randomly and, as expected, when a small asymmetry is taken into account, the patient spends more time in the *health* state than in the *no health* state. This means that there is a finite probability that for any initial condition nearby *x*_1_ (or *x*_2_) the patient will jump across *x*_0_ at a finite time *τ* (i.e., time duration in a given state), switching from one state to the other. 

As seen from Figure 4, the duration time of each state, *τ*, is a random variable. The mean value of *τ* in the *health* state (*x*_1_) can be estimated [6] and is given by

(5) $$\begin{array}{c} \tau_{x1}\approx e^{2\Delta V_{1}/\varepsilon } \end{array}$$

while in the *no health* state (*x*_2_) is

(6) $$\begin{array}{c} \tau_{x2}\approx e^{2\Delta V_{2}/\varepsilon }, \end{array}$$

where *e* is the Nepero number. It is interesting to note that, knowing the mean values of duration times in the *health* and *no health* states, it is possible to estimate the ratio between Δ*V*_1_ and Δ*V*_2_, say the asymmetry of the double-well. 

For the dataset discussed in the previous section, we have *τ*_*x*1_ ≈ 100 weeks and *τ*_*x*2_ ≈ 4.3 weeks. On the other hand, taking the logarithm of (5) and (6), and performing their ratio, we get

(7) $$\begin{array}{c} \frac{\Delta V_{1}}{\Delta V_{2}}\approx \frac{\log\left(\tau_{x1}\right)}{\log\left(\tau_{x2}\right)}. \end{array}$$

Thus, the ratio of the logarithms of duration times in the states *x*_1_ and *x*_2_ provides an estimate of the ratio between the barriers that must be overcome to move from one state to the other. By considering the duration times *τ*_*x*1_ and *τ*_*x*2_ averaged over all the cases of 70 patients it is found Δ*V*_1_/Δ*V*_2_ ≈ 3.1. This implies that the well in *x*_1_ is about three times deeper than that in *x*_2_.

We point out that the ratio between the depths of the wells can be estimated for each patient, provided that the mean duration times of the two states are known from the clinical history. As an example, we have estimated such a ratio for the three sample patients considered in Figure 2. We found

 
*τ*_*x*1_ ≈ 117.7 weeks, *τ*_*x*2_ ≈ 1.5  weeks, Δ*V*_1_/Δ*V*_2_ ≈ 11.8, for the patient no. 23;
 
*τ*_*x*1_ ≈ 54.0 weeks, *τ*_*x*2_ ≈ 2.1  weeks, Δ*V*_1_/Δ*V*_2_ ≈ 5.3, for the patient no. 32;
 
*τ*_*x*1_ ≈ 47.0 weeks, *τ*_*x*2_ ≈ 4.3  weeks, Δ*V*_1_/Δ*V*_2_ ≈ 2.7, for the patient no. 53.

**Figure 8:**
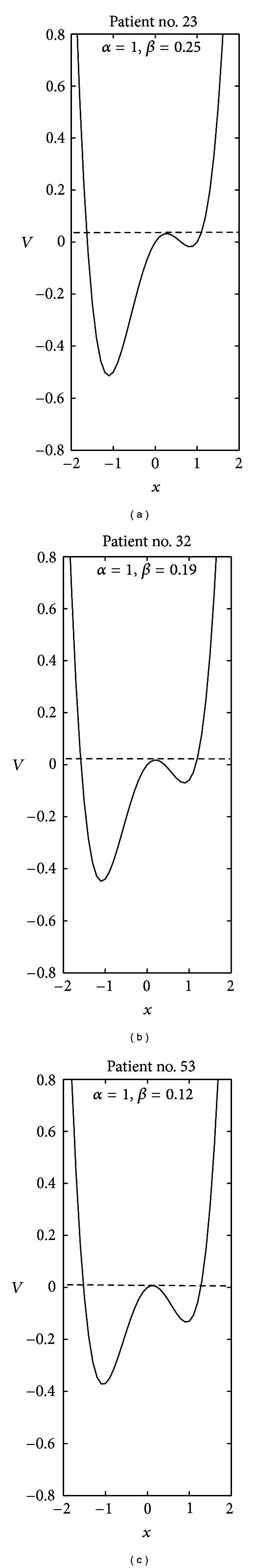
Potential *V* for three sample patients. As an example, the three patients in Figure 2 are considered (no. 23, no. 32 and no. 53 in our dataset) and the mean duration of their relapses and remissions estimated. These values are then used to compute the ratio of the potential barriers Δ*V*_1_/Δ*V*_2_ according to (7) (for the estimates see Model Solutions). The potential *V* for each patient ((a)–(c)) is given by (3) setting *α* = 1 and choosing *β* so that the two wells are asymmetric in the predetermined ratio. In particular, it is found (a) *β* = 0.25, (b) *β* = 0.19, and (c) *β* = 0.12. Dashed line denotes the upper bound of the potential barriers.

At this stage, the potential *V* associated with each patient can be estimated by fixing *α* = 1 and changing *β* so that the two wells are asymmetric in the predetermined ratio. Plots of the three potentials are shown in Figure 8. By assuming that random stimuli of the same variance are applied to the three patients, the shapes of the potentials reflect the differences in the duration times of health and disease states observed in Figure 1. The first potential has a very deep well associated with the *health* state and a very shallow well associated with the *no health* state: this configuration lets the patient be affected by a few disease attacks of short duration. Given the same observation period, the patient no. 32 is expected to have more attacks compared to the previous patient due to the difference in the depth of the wells. Lastly, the patient no. 53 when compared to the patient no. 32 should experience disease events lasting more time because of the deeper well associated with the *no health* state. 

## 4. Discussion

The contribution of the heritable or non-heritable factors that drive MS onset and course appear uniformly too small to explain its etiology, variable severity, and erratic manifestations. We present a mechanistic, stochastic model that describes how the effects of the above forces may be randomly amplified over time. Given a time of observation *T* (e.g., the average lifespan of a person), patients may experience a transition between the two states with a mean time *τ*. Thus, the probability to observe a transition requires that *T* is much longer than *τ*. However, since *τ* is randomly distributed, by mere chance, the transition may or may not occur within the observation time *T*. Within this approach, time is conceived not only as a random variable characterizing disease events but also as the “observation time” needed to detect the onset of the disease in “any susceptible” patient. Stochastic noise in gene expression, through its pervasive effects on virtually all biological processes, may be the factor that amplifies and reshapes the deterministic effects of genetic and environmental risk factors.

The model is in accord with a variety of data in MS and may be general enough to explain features of other complex traits. First, time is a key variable in this model but also in experimental autoimmunity where there is evidence of a multistep process made of subtle alterations—resulting from quantitative trait loci variations—that may accumulate with time and end up in susceptibility when a threshold for the occurrence of the disease is passed [21, 22]. Furthermore, defects of the immune response that originate from predisposing genetic variants are present before any onset of autoimmunity [23]. These relatively recent observations are in accord with classical epidemiological data suggesting that fairly long time intervals are a necessary factor for the development of MS [24, 25]. Lastly, time itself remains an essential component of the diagnostic algorithm of MS [26]. It is therefore not surprising that, if time is a crucial factor to understand the nature of the disease in diagnostic terms, it may also be instrumental for the pathogenetic understanding of the process. 

Second, our model links the degree of genetic variation with the probability that the transition between health and disease states occurs over time. In fact, MS risk alleles influence age of onset [27] and correlation of age of onset in MS relative pairs is proportional to genetic sharing [28]. Interestingly, the model applies also when individuals share genetic protection as witnessed by our observation of an underrepresentation of MS among Italian twins, possibly due to protective factors shared by twins in a Mediterranean area [29]. Furthermore, as expected, a link between genetic variation and transition between the two states over time seems to be maintained during the disease course: with one exception [30], the vast majority of the studies on the natural history of MS has shown that, while the occurrence of relapses is unpredictable, their frequency and severity tend to be conserved as a longer first interattack interval is associated with a better prognosis [31–34]. 

While this is the first time that a model combining deterministic factors and stochastic forcing is applied to the interpretation of a multifactorial disease, determinism and stochasticity have been already shown to fit basic biological mechanisms ([8–12] and references therein). Here, stochasticity is beneficial to “the individual, the colony, or the species” [35] in that it may exert a physiological regulatory role. It may therefore be feasible to seek strategies that try to revert a “pathogenic stochasticity” to its original physiologic role. In the stochastic resonance, introduced to explain Earth's glaciations [5] and sometimes erroneously associated with the random transitions illustrated above, the model solution is driven by the cooperative effect of the stochastic perturbation and the small periodic forcing, leading to a flip from one state to the other with the frequency proper of the external periodic forcing; the small stochastic perturbation provides the energy necessary for the transition, while the periodic forcing sets the periodicity of the transitions. The stochastic resonance occurs for low frequency of the periodic forcing, say for long time periods. For climate changes that induce glaciation cycles [6], such a period is set to 100000 years that describes the long-term variation of the energy input prescribed by astronomical theory. In the case of relapsing-remitting MS, as clinical data suggest, there is not a typical periodicity of relapses induced by some external factors. However, we can imagine to exploit a small external cyclic component in order to “stabilize” the solution around a given state (the *health* state) “deamplifying” it with some short periodicity, contrary to what happens in the stochastic resonance, in order to prevent relapses. A nonmutually exclusive approach may be to decrease the variance of the noise *ε* (possibly acting on fluctuations of gene expression).

This study offers a new interpretative key of the events that characterize the clinical course of MS. By inference, it also suggests that time and noise amplify and adjust the deterministic influence of genes and environment on the disease. It will not be easy to obtain direct experimental evidence for this model at the disease level. However, basic science data—increasingly supporting the notion that noise provides critical functions at the cell level—suggest that this effort will probably be a rewarding one. Thus far the model may contribute to the understanding of phenotypic variation in health and disease states.

## 5. Conclusion

Despite the advent of genomewide association studies (GWAS; an approach to look for associations between hundreds of thousands genetic variations and diseases), many questions remain about the causative mechanisms of multifactorial diseases. In particular, the effect size of the genetic variants identified through these studies explains relatively little of the heritability (the proportion of total variance in a population for a particular measurement, taken at a particular time or age, that is attributable to variation in genetic values) of most complex traits. So far, the same is true for environmental risk factors. Multiple sclerosis is one of the diseases that best exemplify this key problem. By observing the course of this disease in its relapsing-remitting phase we found that a mechanistic model with a random forcing describes the main features of the disease course. If the model is applied to the events that precede the disease (i.e., those that derive from the effects of the genetic and environmental risk factors), it may explain how relatively small effects may be amplified by comparably small random perturbations. As such, the model may inform the design of future studies on gene-environment interactions in multifactorial diseases.

## Figures and Tables

**Table 1:** Disease characteristics and parameters of the mechanistic stochastic model. The table summarizes analogies and links between MS characteristics (left column) and model parameters in (4), in the right column.

Disease characteristics during the relapsing-remitting phase	Mechanistic, stochastic model
Two states are observed that we referred to as *health* and *no health*.	*V*(*x*): double-well potential (two minima associated with the two states).
The disease develops in patients with a certain degree of heritable and nonheritable risk.	*α*: control parameter that sets the barrier to overcome for going from one state to the other.
It is found that the time spent in the state of *health* is much longer than in the *no health* state.	*β*: asymmetry parameter of the potential.
Random variability of biological processes (i.e. randomness in transcription and translation leading to cell-to-cell variations) [8].	*ε* ^1/2^ *dw*: stochastic perturbation with zero mean and variance *ε*.
Genetic background and environmental factors alone are not enough to trigger the transitions between the two states.	The noise provides the required energy for the random transitions.
